# Miniature Wide-Band Noise-Canceling CMOS LNA †

**DOI:** 10.3390/s22145246

**Published:** 2022-07-13

**Authors:** David Galante-Sempere, Javier del Pino, Sunil Lalchand Khemchandani, Hugo García-Vázquez

**Affiliations:** 1 Institute for Applied Microelectronics (IUMA), Departament of Electronics and Automatic Engineering, University of Las Palmas de Gran Canaria (ULPGC), Campus Universitario de Tafira, 35017 Las Palmas de Gran Canaria, Spain; jpino@iuma.ulpgc.es (J.d.P.); sunil@iuma.ulpgc.es (S.L.K.); 2 Instituto de Astrofísica de Canarias (IAC), 38205 San Cristóbal de La Laguna, Spain; hugo.garciavazquez@iac.es

**Keywords:** RFIC, wide-band, low noise amplifier, current conveyor, noise canceling, CMOS

## Abstract

In this paper, a wide-band noise-canceling (NC) current conveyor (CC)-based CMOS low-noise amplifier (LNA) is presented. The circuit employs a CC-based approach to obtain wide-band input matching without the need for bulky inductances, allowing broadband performance with a very small area used. The NC technique is applied by subtracting the input transistor’s noise contribution to the output and achieves a noise figure (NF) reduction from 4.8 dB to 3.2 dB. The NC LNA is implemented in a UMC 65-nm CMOS process and occupies an area of only 160 × 80 μm^2^. It achieves a stable frequency response from 0 to 6.2 GHz, a maximum gain of 15.3 dB, an input return loss (S11) < −10 dB, and a remarkable *IIP*3 of 7.6 dBm, while consuming 18.6 mW from a ±1.2 V DC supply. Comparisons with similar works prove the effectiveness of this new implementation, showing that the circuit obtains a noteworthy performance trade-off.

## 1. Introduction

A low-noise amplifier (LNA) is one of the key elements in a wide-band receiver. As the first element in the receiving chain, its noise figure (NF) and gain have a greater impact than other modules on the overall performance. Source impedance matching is also required to limit reflections. Conventional broadband LNA designs, such as resistive-loaded common source (CS) or common gate (CG) amplifiers, have proven difficult to meet the above requirements [1]. On the other hand, amplifiers employing a global negative feedback can achieve low NF with good input matching, but they are prone to becoming unstable [2]. Other alternatives, such as distributed amplifiers (DAs) [3] or cascode amplifiers with LC broadband input-matching networks [4,5,6], provide good impedance matching and high gain in a larger frequency range, but they need several inductors. Another topology used to design broadband LNAs is based on the use of current conveyors (CC) [7,8,9]. Although they have many advantages, such as good input matching, high linearity, and low power consumption, they suffer from the drawback of having a relatively high NF. In this paper we explore the use of noise-canceling techniques [10,11,12] to obtain a significant reduction in the NF of CC-based broadband LNAs. The goal is to present an amplifier topology that combines the advantages of a CC-based circuit with the possibility of reducing the NF at the output of the circuit.

The organization of the paper is as follows. In Section 2, a wide-band amplifier based on CC is discussed. In Section 3, the noise-canceling technique of a CG amplifier is presented. The CC-based amplifier with noise cancelation is introduced in Section 4. Section 5 reports the simulation results of the proposed LNA implemented in a UMC 65-nm CMOS process. Finally, conclusions of this work are drawn in Section 6.

## 2. CMOS Current Conveyor Based LNA

**Figure 1:**
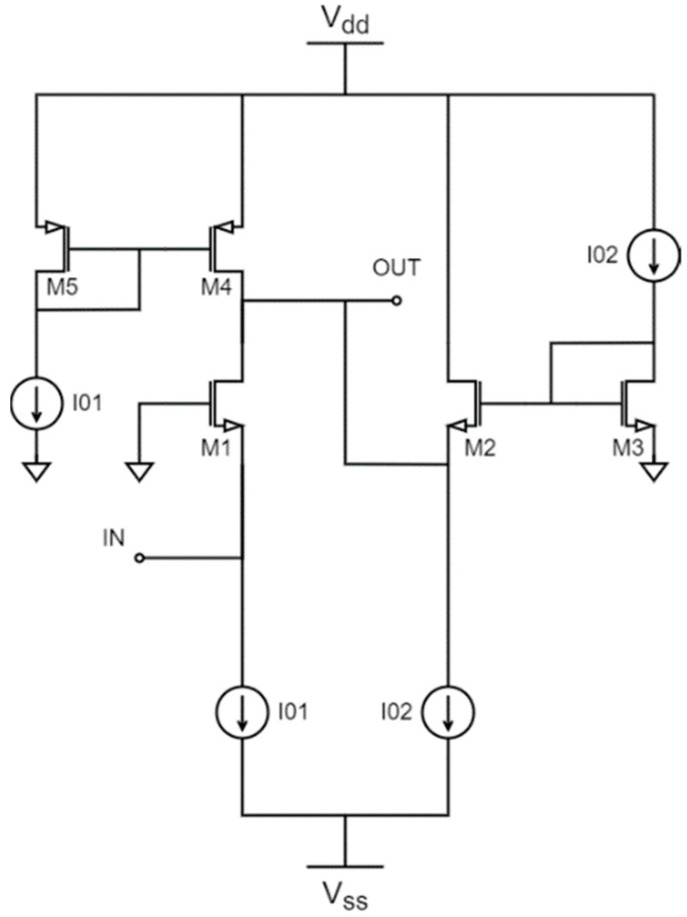
CMOS LNA based on current conveyors with ideal current sources.

The basic topology of a CC-based amplifier using CMOS technology is shown in Figure 1. It consists of an input CG gain stage (M1), followed by a source follower stage (M2) [8].

The DC gain (*G*), bandwidth ($f_{-3\mathrm{dB}}$), and noise factor (*F*) can be calculated as shown by (1), (2), and (3), respectively, where *g_m_*_1_ and *g_m_*_2_ are the M1 and M2 transconductances, *C_T_* represents the total parasitic capacitance at the output node, and γ is the excess noise factor, which is a constant that depends on the transistor size.

(1) $$G=\frac{g_{m1}}{g_{m2}}$$

(2) $$f_{-3\mathrm{dB}}=\frac{g_{m2}}{{2\pi C}_{T}}$$

(3) $$F=1+\gamma \left(1+\frac{g_{m2}}{g_{m1}}+\frac{g_{m4}}{g_{m1}}\right)$$

The circuit’s input and output impedances can be easily computed as

(4) $$Z_{in}=\frac{1}{g_{m1}},$$

(5) $$Z_{out}=\frac{1}{g_{m2}}.$$

There is a trade-off between *g_m_*_1_ and *g_m_*_2_ and, consequently, between the transistor sizes and bias currents. Current I_01_ affects the gain through *g_m_*_1_, but has no effect on the bandwidth, while I_02_ controls the gain and bandwidth through *g_m_*_2_ (if I_02_ is increased, the bandwidth increases, but the gain is decreased). On the other hand, when *g_m_*_1_ increases, *F* decreases, as shown in (3). This can be done by increasing the size of M1 or the current I_01_. Likewise, if M2 and M4 are either smaller or biased with a lower I_02_, *F* decreases. Finally, the input and output impedances are directly related to their associated transistors and bias currents. For example, through I_01_, *Z_in_* can be matched to the source impedance without resorting to matching networks, allowing for a much smaller circuit when compared to conventional topologies. This is one of the main advantages of this approach. Following this discussion, the bias currents I_01_ and I_02_ are selected as 1 mA and 200 µA, respectively, to maintain a reasonable power consumption.

**Figure 2:**
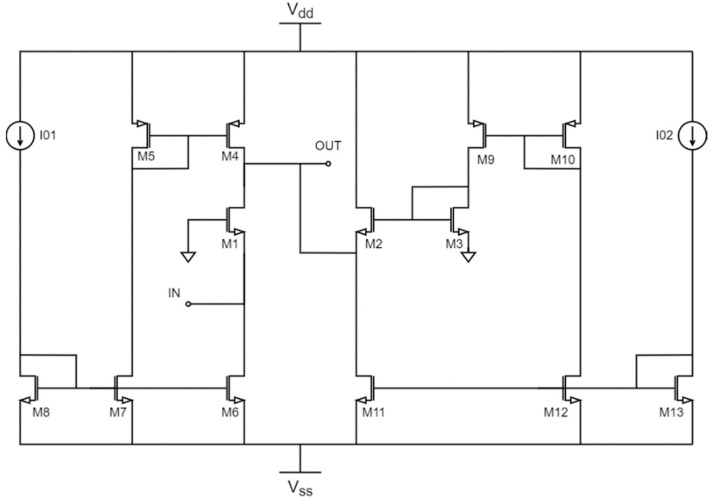
CMOS LNA based on current conveyors with current mirrors as current sources.

The complete implementation of the circuit schematic is shown in Figure 2, where the ideal current sources have been replaced by current mirrors. The high number of transistors certainly affects the performance of the circuit, especially bandwidth, noise, and power consumption. Due to the low output conductance of transistors in deep submicron technologies, the input impedance also deviates from the conventional 1/*g_m_* value. This, rather than being a problem, can be useful to isolate the input-matching condition from the noise-canceling one, providing a degree of freedom in the input-matching design [12].

## 3. Noise-Canceling Technique Applied to a CG Stage

**Figure 3:**
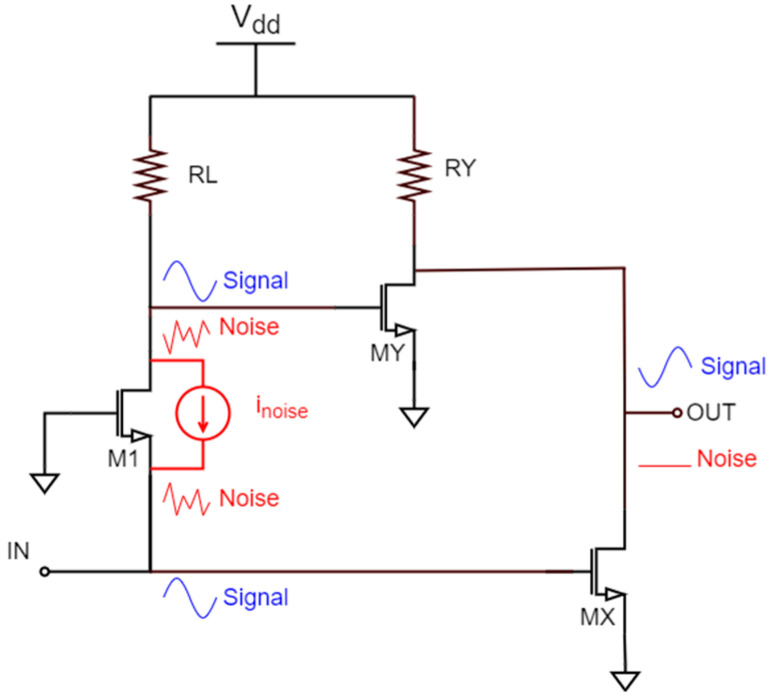
Common-gate amplifier with noise canceling.

**Figure 4:**
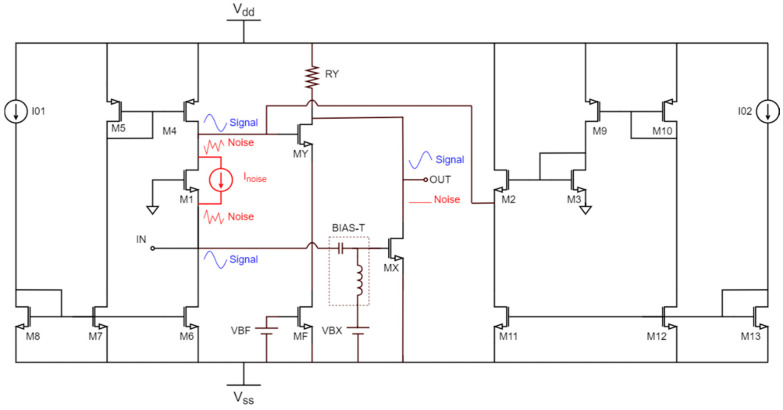
Proposed LNA based on current conveyors with noise canceling.

Equation (3) shows that the input common-gate transistor (M1) is the main noise contributor. Various architectures can be found in the literature to cancel the noise of this topology [10,11,12]. The simplified diagram of a common-gate stage with noise cancelation is depicted in Figure 3. With this technique, the noise of the input transistor passes through two different paths (transistors MX and MY), and is canceled at the output, while the input signal is boosted. This can be seen better in the inset of Figure 4, where the noise and the signals are plotted on the schematic. Transistor M1 is the main noise contributor, and a noise source (i_noise_) represents its contribution in the circuit. This source generates a voltage at the source of M1 and a fully correlated voltage at the drain with the same magnitude and opposite sign. The noise reaches the output through two different paths, and thanks to the CS inverting stages composed by transistors MX and MY, it is canceled at the output. In contrast, the input signal is amplified through the same paths thanks to the CS-CG inverting amplifier (transistors M1 and MY) and the CS inverting amplifier (MX) and is amplified at the output instead of being canceled.

To achieve perfect noise cancelation, the two noise paths should have the same gain. Therefore, transistors MX and MY must be independently biased. It is important to note that transistors MX and MY also introduce noise into the circuit, so they must be carefully designed. Thus, the key to achieve a low overall NF has now shifted to a low noise implementation in the noise-canceling stage.

## 4. LNA Based on CC with Noise Canceling

To better understand the improvements introduced by the noise-canceling technique, two different implementations were developed with the same sizing and bias currents. On the one hand, a current conveyor-based LNA is implemented following the schematic depicted in Figure 2. On the other hand, a second implementation of the LNA applying the noise cancelation technique is developed following the schematic shown in Figure 4.

The circuits are developed using UMC 65-nm CMOS technology with a ±1.2 V DC supply voltage. A summary of the device sizing information is given in Table 1, where the length of all the MOSFETs is fixed to the minimum allowed value of 65 nm. Note that the bias currents I_01_ and I_02_ are selected as 1 mA and 200 µA, respectively, and the DC voltages V_BF_ and V_BX_ are 0.35 V each. In addition, the resistor R_Y_ is selected as 220 ohms. The schematic of the proposed noise-canceling LNA based on CC is presented in Figure 4. This circuit combines the CC-based approach shown in Figure 2 with the noise-canceling technique presented in Section 3. As explained above, the input transistor generates a noise contribution of equal magnitude but opposite phase at its drain and source terminals. These two noise signals pass through two inverting paths and are canceled at the output. On the contrary, the input signal has the same sign on the drain and source terminals of the input transistor, so it passes through the two inverting paths, and it is added at the output. In the design of this circuit, the size of the input stage should be determined to obtain a good broadband input impedance match. The noise-canceling stage must be designed to achieve broadband noise cancelation while introducing as little noise as possible and degrading gain, bandwidth, power consumption, and input matching as little as possible. Using a CC-based LNA as the input stage instead of a simple CG stage gives a degree of freedom in satisfying the input-matching conditions.

## 5. Simulation Results and Analysis

**Figure 5:**
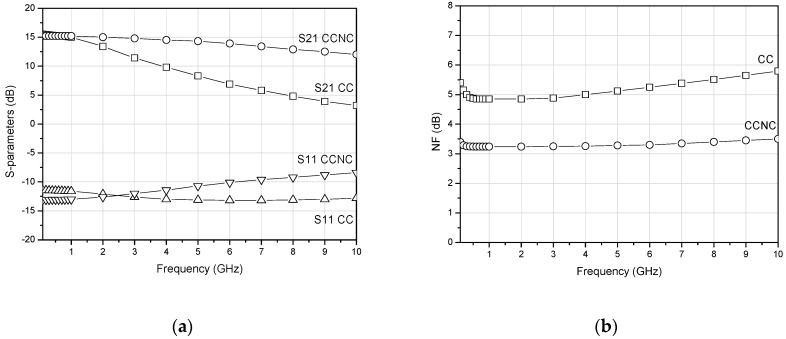
Simulation results of (**a**) the S–parameters and (**b**) the NF of the LNA based on current conveyors (CC) and the proposed LNA based on current conveyors with noise cancelation (CCNC).

The simulated performance comparison of the LNA based on CC (CC) and the proposed LNA based on CC with noise cancelation (CCNC) is shown in Figure 5. The proposed technique reduces the noise figure from 4.85 dB to 3.25 dB at 1 GHz. At frequencies below 6 GHz, both circuits present a reasonable input matching, with an input return loss (S11) < −10 dB. However, at higher frequencies the input matching deteriorates slightly. This can be solved by simply increasing the bias current I_02_, but at the expense of increasing the power consumption.

**Figure 6:**
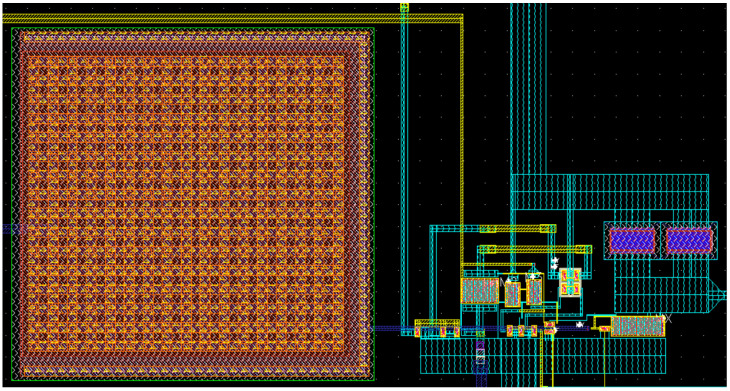
Physical chip layout of the proposed LNA based on current conveyors with noise cancelation (occupied area: 160 × 80 μm^2^).

The layout of the proposed LNA is shown in Figure 6. To reduce the circuit area, the BIAS-T inductor needed to bias the MX transistor is replaced by a large resistor, which is implemented in practice by the large leakage resistance of a reverse-biased p-n junction of a diode-connected MOS transistor operating in the cutoff region (2 × 0.5 µm). Note that the exact value of this resistance or its temperature and voltage dependence are not relevant, provided that it remains large enough not to influence the circuit operation at the lowest frequency required. As no inductor is present, the LNA core occupies an area of only 160 × 80 μm^2^, which is among the smallest designs available in the literature.

**Figure 7:**
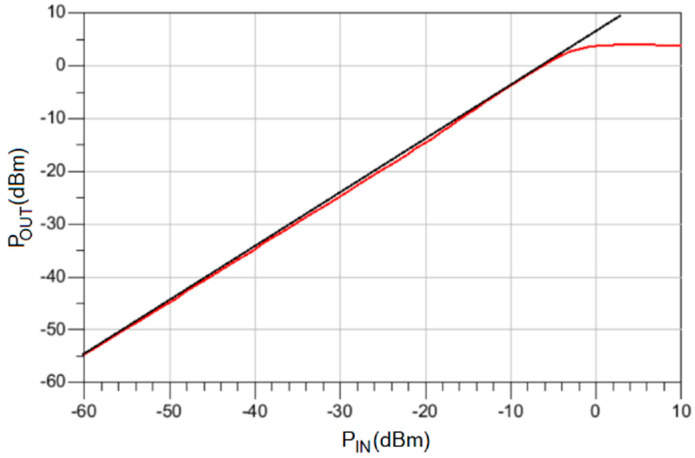
Simulated output power (red line) as a function of the input power to calculate the 1-dB compression point. The black line represents the ideal response without distortion.

Linearity is a very important feature of an LNA since a higher 1-dB compression point means a larger capability of receiving weak signals in the presence of strong ones. This technique presents the advantage of significantly boosting the circuit’s linearity, since the same mechanism leading to noise cancelation can also cancel partially the nonlinear distortions [13]. The simulation results shown in Figure 7 demonstrate an input 1-dB compression point of –2 dBm. Finally, the power dissipation of the circuit is 18.57 mW, with a DC supply of ±1.2 V.

The performance of the proposed LNA is compared in Table 2 with recently reported LNAs available in the literature. To fairly compare the proposed CCNC LNA with the other designs, we have defined the following figure of merit (*FoM*), which considers a positive contribution of amplifier gain, bandwidth, and *IIP3*, and a negative contribution of power consumption, noise factor, and active area:

(6) $$FoM=\frac{Gain\left(\mathrm{abs}\right)\times BW\left(\mathrm{GHz}\right)\times IIP3\left(\mathrm{mW}\right)}{P{\;}_{DC}\left(\mathrm{mW}\right)\times \left(F-1\right)\times area\left({\mathrm{mm}}^{2}\right)}.$$

The proposed CCNC LNA presents the highest *FoM* value among the reported designs. This is because the proposed technique achieves a high bandwidth and *IIP*3 values using a very small area, while the gain and NF are within the average. The price to pay is a slightly higher power consumption than most of the other designs in the comparison.

## 6. Conclusions

A wide-band CMOS noise-canceling current conveyor-based LNA is proposed in this paper and is implemented in a standard 65-nm CMOS process. The design exploits a noise-canceling technique consisting of an amplifying stage based on a CC that provides input impedance matching, and a noise-canceling stage composed of two transistors in CS configuration that subtracts the input transistor noise contribution while adding the signal contributions. The simulation results show that the proposed CCNC LNA achieves wide-band input impedance matching (0–6.2 GHz), with high gain (15.3 dB) and low noise (4.8 dB), in a very small area (160 × 80 μm^2^). The circuit presents a power dissipation of 18.57 mW from a DC supply of ±1.2 V. In addition, the proposed noise-canceling technique also improves the linearity because it is capable of partially canceling nonlinear distortions. The proposed circuit achieves a remarkable *IIP*3 of 7.6 dB and an input P_1dB_ of –2 dBm. The price to pay with this approach is the additional power dissipation introduced by the auxiliary amplifier in the noise-canceling path. As shown in the comparison with similar works, the proposed circuit presents the highest *FoM* value among the reported designs.

## Figures and Tables

**Table 1:** Device sizes summary of the implemented LNAs.

M1	M2	M3	M4	M5	M6	M7	M8
20 × 5 μm	5 × 5 μm	2 × 0.5 μm	2 × 0.5 μm	2 × 0.5 μm	2 × 0.5 μm	2 × 0.5 μm	2 × 0.5μm
**M9**	**M10**	**M11**	**M12**	**M13**	**MX**	**MY**	**MF**
2 × 0.5 μm	2 × 0.5 μm	2 × 0.5 μm	2 × 0.5 μm	2 × 0.5 μm	30 × 4 μm	2 × 0.5 μm	2 × 0.5 μm

**Table 2:** Performance summary of the proposed LNA and similar works available in the literature.

Parameter	Soleymani [14]	Iji [15]	Baumgratz [16]	Wang [17]	Liu [18]	Baumgratz [19]	Hsieh [20]	Farzaneh [21]	Farzaneh [21]	This Work CCNC	This Work CC
Year	2020	2012	2017	2017	2016	2019	2020	2020	2021	2022	2022
Tech. (nm)	180	250	130	180	65	130	180	180	90	65	65
S_11_ (dB)	<−21	<−10	<−10	N/A	N/A	<−10	<−10	<−10	<−10	<−10	<−10
BW (GHz)	1.1	1.1	2.9	0.3875	2	3.1	0.1	3.8	4.7	6.2	6
Gain (dB)	20.2	13.8	10	15	24	20	10	22	21	15.3	15.3
*IIP*3 (dBm)	−9.5	−12	−10	32 *	11.6	−11.1	0	−4	−4	7.6	7.6
Power (mW)	8	1.8	15.6	43	3.48	19	0.6	13	8.8	18.6	18.6
NFmin HG (dB)	1.68	2.3	4.9	15	24	3.4	4	3.25	3.2	3.2	4.8
Area (mm^2^)	0.0158	1	0.15	0.1	0.01	0.15	0.9	0.0258	0.014	0.0128	0.0128
*FoM*	21.16	0.27	0.19	26.23	52.62	0.71	0.39	51.00	156.44	797.27	415.99
Sim./Meas.	Sim.	Sim.	Meas.	Sim.	Meas.	Meas.	Meas.	Sim.	Sim.	Sim.	Sim.

N/A, Not Available; * OIP3.

## Data Availability

Not applicable.
